# The cross-sectional shape of the fourfold semitendinosus tendon is oval, not round

**DOI:** 10.1186/s40634-016-0063-3

**Published:** 2016-10-12

**Authors:** Takeshi Oshima, Junsuke Nakase, Hitoaki Numata, Yasushi Takata, Hiroyuki Tsuchiya

**Affiliations:** Department of Orthopaedic Surgery, Graduate School of Medical Science, Kanazawa University, 13-1 Takara-machi, Kanazawa, 920-8641 Japan

**Keywords:** Anterior cruciate ligament reconstruction, Fourfold semitendinosus tendon, Rounded rectangular tunnel, Cross-sectional shape

## Abstract

**Background:**

The looped side of the semitendinosus tendon (ST) graft (i.e., the side inserted into the femoral tunnel during anterior cruciate ligament reconstruction) appears to be oval rather than round. The purpose of this study was to investigate the cross section of the fourfold semitendinosus tendon graft and, more specifically, the differences in pressure exerted by a rounded rectangular tunnel versus a round femoral tunnel.

**Methods:**

Seven STs were harvested from cadaveric knees and a fourfold ST graft was made. Aluminum cubes with round or rectangular tunnels containing four-way pressure-sensitive conductive sensors (vertically and bilaterally) were used. The area of both cubes was the same. The graft was inserted into the tunnels 15 mm from the looped edge. After measuring pressure, the graft was fixed using ultraviolet-curing acrylic resin and was cut at 7.5 mm and 15 mm from the lapel edge. The area, axes for the best fitting ellipse of the cross-section, and ellipticity of the axes were measured.

**Results:**

In the round tunnel, the mean contact pressure was 287.0 ± 136.7 gf at the bilateral sensor; there was no contact pressure detected by the vertical sensor. In the rounded rectangular tunnel, the mean contact pressure was 260.9 ± 186.4 gf at the bilateral sensor and 352.9 ± 49.5 gf at the vertical sensor. Ellipticity was 1.25 ± 0.13 at 7.5 mm, and 1.17 ± 0.07 at 15 mm from the lapel edge of the graft.

**Conclusions:**

The cross-sectional shape of the fourfold ST graft was not round, but oval. Moreover, the rounded rectangular tunnel was more fitted to the graft than the round tunnel.

## Background

Annually, 100,000 to 200,000 anterior cruciate ligament (ACL) knee injuries occur in the United States (Beynnon et al. 1997). It is well kn own that conservative treatments for ACL injury yield unsatisfactory outcomes, especially for young patients who wish to maintain an active lifestyle. Therefore, ACL reconstruction remains a treatment of choice and various improvements to the procedure have been accomplished since it was first performed.

Recently, there has been general agreement that tunnel the positions within the anatomical insertion points of the ACL are fundamental to successful ACL reconstruction and long-term stability (Kamath et al. 2011; Marchant et al. 2010). Some clinical studies indicate that non-anatomical ACL graft placement is the most common technical error that subsequently leads to recurrent instability after reconstruction (Kamath et al. 2011; Marchant et al. 2010). Therefore, one of the most critical factors for successful ACL reconstruction is proper placement of the ACL graft (Khalfayan et al. 1996; McConkey et al. 2012), although this topic remains controversial. The arthroscopic anatomical double-bundle ACL reconstruction technique was first reported in 2004 by Yasuda et al. (Yasuda et al. 2004). Since that time, several studies have reported that the anatomical double-bundle technique provides more stable anterior–posterior translation and restores more rotational stability as compared with conventional single-bundle ACL reconstruction (Aglietti et al. 2010; Colombet et al. 2007; Yagi et al. 2007). However, there are some concerns about double-bundle ACL reconstruction. One is the need to drill four independent tunnels, which increases the risk of incorrect tunnel placement, and several authors have reported significant tunnel widening after the procedure (Siebold 2007). Siebold and Zantop postulated that a potential indication for double-bundle ACL reconstruction is tall patients with large insertion zone (Siebold & Zantop 2009). Furthermore, double-bundle ACL reconstruction creates more extensive bone loss and requires longer operative times, thereby potentially increasing the difficulty of revision surgery. Therefore, attention has returned to single-bundle reconstruction with grafts that are placed at the center of anatomical footprint. Several recent biomechanical studies have shown that single-bundle ACL grafts placed in the center of their anatomic insertions can restore nearly normal knee kinematics, which is comparable to results achieved with double-bundle ACL reconstruction (Ho et al. 2009; Sastre et al. 2010). Harms et al. showed that single-graft ACL reconstruction performed at the central femoral and tibial ACL attachment sites can restore anterior-posterior translation and tibial rotation motion limits. In addition, rotational knee stability was restored under all simulated pivot-shift testing conditions (Harms et al. 2015). Using a navigation system, Porter et al. showed that “anatomic” single-bundle ACL reconstruction reduces both anterior translation and internal rotation during pivot shifts (Porter & Shadbolt 2014). Clinically, Mayr et al. showed there were no differences in International Knee Documentation Committee subjective and objective scores between patients who underwent anatomic-single bundle ACL reconstruction and those who underwent anatomic double-bundle ACL reconstruction (Mayr et al. 2016). It is widely accepted that the ACL almost attaches posteriorly to the lateral intercondylar ridge, and several anatomical studies have reported that the femoral insertion area is an oval or semilunar shape (Petersen & Zantop 2008; Sasaki et al. 2012). In practice, we have realized that the looped side of the fourfold semitendinosus tendon graft—the side that is inserted into the femoral tunnel—appears to be oval or a rounded rectangle, rather than round. However, to the best our knowledge, no report discusses the cross-sectional shape of the semitendinosus tendon graft scientifically. Furthermore, we hypothesize that the pressure of the graft on the femoral tunnel is more equal in a rounded rectangular tunnel because of the unrounded shape. Thus, the purpose of this study was to investigate the cross section of the fourfold semitendinosus tendon graft and, more specifically, the differences in pressure exerted by a rounded rectangular tunnel versus a round femoral tunnel.

## Methods

**Fig. 1:**
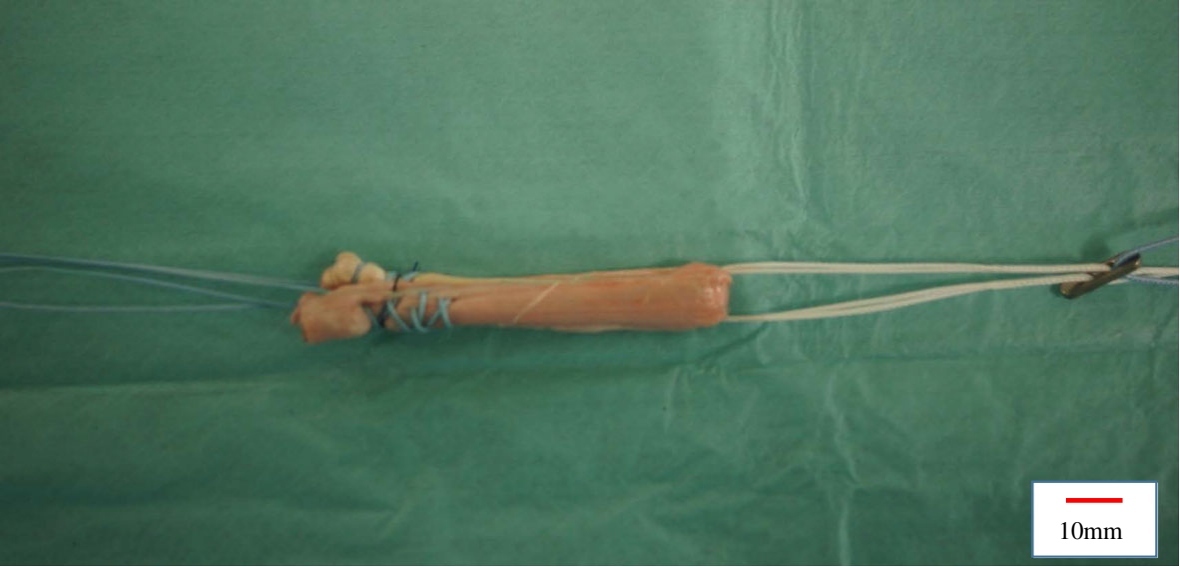
Four-fold semitendinosus tendon graft. A four-fold semitendinosus tendon graft looped over a TightRope

Seven semitendinosus tendons were harvested from seven fresh-frozen cadaveric knees (age: 80.3 ± 7.3 y; height: 166.4 ± 6.0 cm; weight: 57.9 ± 8.4 kg). For a fourfold graft, a minimum length of 25 cm is needed for the semitendinosus tendon, which is normally achieved by cadaveric knees. After removing excess soft tissue, both ends of the double-fold semitendinosus tendon were sutured with a baseball stich using No. 2 FiberWire (Arthrex Co., Ltd., Naples, Florida, USA). Next, the double-fold semitendinosus tendon was looped over TightRope (Arthrex Co., Ltd., Naples, Florida, USA) to make a fourfold ST graft (Fig. 1). The graft tension was set to 30 N. We removed only excess soft tissue, and did not do arbitrary processing.

### Study 1: Measurement of the graft contact pressure

A 2-mm pressure-sensitive conductive rubber sensor (Inaba Rubber Co., Ltd., Osaka, Japan) was used to measure the contact pressure of the graft. In our investigation, the pressure-sensitive conductive rubber sensor exhibited an active area of 1.5 × 20 mm; additionally, the strongest pressure in the active area was measured and the active range of pressure was measured from 0 to 3000 gf.

**Fig. 2:**
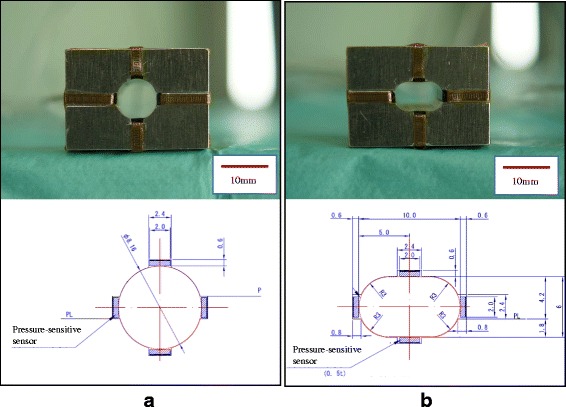
Tunnel models with pressure-sensitive conductive rubber sensors. **a** An aluminum cube with round tunnel (8.16 mm diameter). **b** An aluminum cube with rounded rectangular tunnel (6 × 10 mm). Pressure-sensitive conductive rubber sensors were contained vertically and bilaterally

**Fig. 3:**
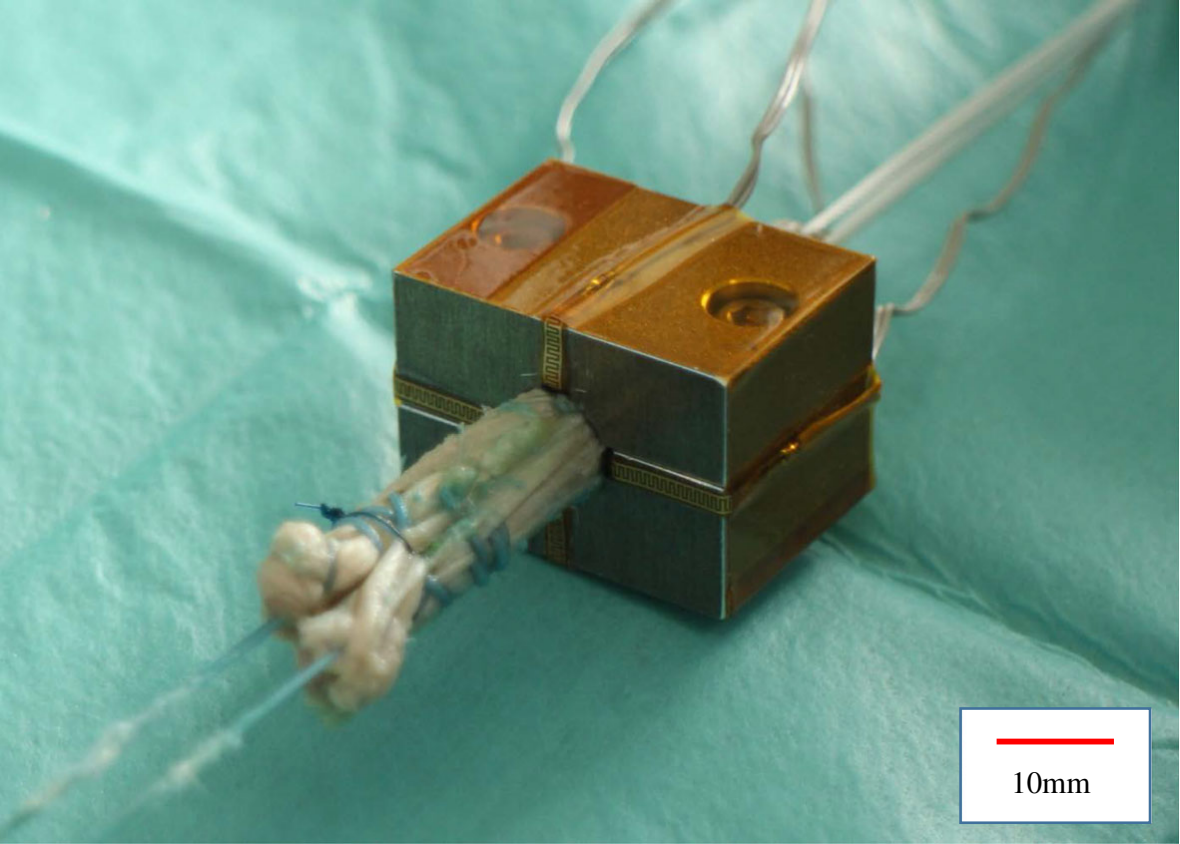
Measurement of the Graft Contact Pressure. The graft was inserted into the tunnels 15 mm from the looped edge and its crease accorded with bilateral sensors

Aluminum cubes with two types of tunnel containing four-way pressure-sensitive conductive rubber sensors (vertically and bilaterally) were created. One tunnel was round (8.16 mm diameter) and another was rounded rectangular (6 × 10 mm) in shape. The area of cross-section was the same for both tunnels (52.3 mm^2^) (Fig. 2). The graft was inserted into the tunnels 15 mm from the looped edge and its crease accorded with bilateral sensors. The pressure was measured three times (Fig. 3). We adopted gram-forece (gf) to the unit of the pressure.

### Study 2: Investigation of the graft cross-section

**Fig. 4:**
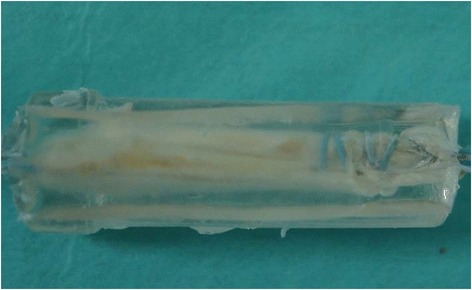
How to fix the graft. **a** The graft was dipped in a mold filled with resin and irradiated with ultraviolet light for three minutes. **b** The fixed graft with Ultraviolet-curing acrylic resin

Ultraviolet-curing acrylic resin (Kiyohara Co., Ltd., Osaka, Japan) and an ultraviolet lamp (wavelength 365 nm; Eiko Co., Ltd., Kanagawa, Japan) were used to fix the graft with 30-N tension. The graft was dipped in a mold filled with resin and irradiated with ultraviolet light for three minutes (Fig. 4). After being fixed, the graft was cut at 7.5 mm and 15 mm from the looped edge using a diamond T-saw (Medtronic Sofamor Danek Co., Ltd., Memphis, TN, USA).

**Fig. 5:**
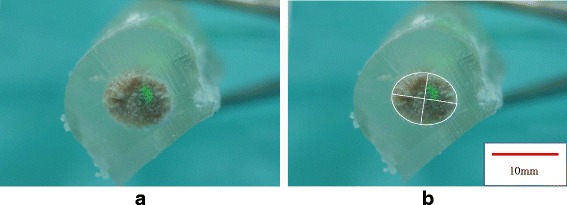
Cross sectional shape of the graft. **a** A cross section of the graft. **b** A best- fitting ellipse of the cross-section was analyzed using image J

Photographs of the cross-sections were taken using a digital camera (Sony SLT-33A; Sony Inc., Tokyo, Japan). The photographs were analyzed using ImageJ 1.50b computer software (National Institutes of Health, Bethesda, MD, USA) to measure the area of the cross-section; the major and minor axes of the best- fitting ellipse of the cross-section; and ellipticity (i.e., the ratio of the major and minor axes of the ellipse; Ellipticity = major axis/ minor axis) (Fig. 5).

### Statistical analysis

Data are presented as mean ± standard deviations, and the significance level was set at *P* = 0.05. Pearson’s correlation coefficient was measured between the graft pressure of each tunnel and the parameters of the cross-section of the graft. Two orthopedic surgeons (T.O. and Y.T.) independently measured the parameters with ImageJ. Each observer performed each measurement three times, with observations being spaced at least 1 week apart. Reliability of the measurements was assessed by examining the interobserver and intraobserver variations with the intraclass correlation coefficient (ICC). An ICC > 0.80 was considered to represent a reliable measurement. The interobserver and intraobserver variations for the measurements were satisfactory, with the mean ICC values being 0.95 and 0.99, respectively.

## Results

In the round tunnel, the mean contact pressure was 287.0 ± 136.7 gf at the bilateral sensor; no contact pressure was detected by the vertical sensor. In the case of the rounded rectangular tunnel, the mean contact pressure was 260.9 ± 186.4 gf at the bilateral sensor and 352.9 ± 49.5 gf at the vertical sensor.

The cross-section of the graft was not round, but oval (Fig. 5). The area was 55.5 ± 3.9 at 7.5 mm and 54.9 ± 5.4 at 15 mm; the major axis was 9.4 ± 0.6 at 7.5 mm and 9.0 ± 0.6 at 15 mm; the minor axis was 7.5 ± 0.5 at 7.5 mm and 7.7 ± 0.3 at 15 mm; and the ellipticity was 1.25 ± 0.13 at 7.5 mm, and 1.17 ± 0.07 at 15 mm from the looped edge of graft (Table 1). The major axis of the best fitting ellipse matched the crease of the graft.

**Table 1:** The contact pressure and the parameters of the cross sections

	Cross-section								Contact pressure			
	7.5 mm from the looped edge				15 mm from the looped edge				Round tunnel		Rounded rectangular tunnel	
Cadaver no.	Area (mm^2^)	Major (mm)	Minor (mm)	Ellipticity	Area (mm^2^)	Major (mm)	Minor (mm)	Ellipticity	Vertical (gf)	Bilateral (gf)	Vertical (gf)	Bilateral (gf)
1	59.5	10.3	7.4	1.07	59.7	9.9	7.7	1.10	0	354.7	390.8	415.3
2	59.5	9.0	8.4	1.22	60.1	9.8	7.8	1.14	0	366.5	298.7	268.4
3	57.4	9.3	7.9	1.17	55.9	9.0	7.9	1.13	0	271.4	353.0	350.3
4	55.8	9.5	7.5	1.27	53.2	8.8	7.7	1.15	0	410.0	341.0	352.0
5	54.8	10.0	7.0	1.21	59.5	9.1	8.3	1.14	0	341.5	442.2	440.4
6	52.9	9.1	7.4	1.44	49.7	8.5	7.5	1.29	0	265.0	309.0	0
7	48.6	8.6	7.2	1.40	46.4	8.2	7.2	1.27	0	0	335.6	0
Mean	55.5	9.4	7.5	1.25	54.9	9.0	7.7	1.17	0	287.0	352.9	260.9
SD	3.9	0.6	0.5	0.05	5.4	0.6	0.3	0.03	0	136.7	49.5	186.4

There were moderate to strong correlations between the area and the mean contact pressure at the vertical sensor of the rounded rectangular tunnel (*r* = 0.805, *p* = 0.029), between the area and the mean contact pressure at the bilateral sensor of the rounded rectangular tunnel (*r* = 0.895, *p* = 0.007), and between the minor axis and the mean contact pressure at the vertical sensor of the rounded rectangular tunnel (*r* = 0.754, *p* = 0.050) (Table 2).

**Table 2:** Pearson’s correlation coefficient between the graft pressure of each tunnel and the parameters of the cross-section of the graft

	Area	Major axis	Minor axis
Round tunnel	0.495	0.400	0.398
Bilateral sensor	(*p* = 0.259)	(*p* = 0.374)	(*p* = 0.376)
Rounded rectangular tunnel	0.805	0.563	0.754
Vertical sensor	(*p* = 0.029)	(*p* = 0.188)	(*p* = 0.050)
Rounded rectangular tunnel	0.895	0.714	0.702
Bilateral sensor	(*p* = 0.007)	(*p* = 0.071)	(*p* = 0.079)

## Discussion

The core finding of our study was that the cross-section of the femoral insertion side of the graft was not round, but oval (ellipticity was 1.25 ± 0.13 at 7.5 mm and 1.17 ± 0.07 at 15 mm from the edge of graft, respectively). Moreover, contact pressure existed more equally for the rounded rectangular tunnel versus the round tunnel. These results suggest that although semitendinosus tendon is “soft” tissue, a rounded rectangular tunnel is more suitably fitted compared with a round tunnel of the same area.

Lee showed that the cross sectional area of graft in the tunnel by using MRI (Lee et al. 2015). However, to our knowledge, there was no report that directly show cross sectional shape of the graft. This is the first study to evaluate cross-sectional shape and the relationship with contact pressure.

The other side of looped side were divided into two ways and became slightly thick because they were sutured with FiberWire. Therefore there is the difference of ellipsticity according to the length from the edge of the graft.

Since tendon grafts become anchored into bone with Sharpey-like fibers (Oguma et al. 2001; Tomita et al. 2001), a close fit to the bone tunnel may be advantageous in graft anchoring by avoiding the inflow of joint fluid. Because the semitendinosus tendon is soft tissue, it is generally thought that a semitendinosus tendon graft can change the cross sectional shape to fit to the round tunnel and prevent the inflow of joint fluid (Toritsuka et al. 2003). The present study showed that even though the cross-sectional areas of cadavers 1 to 5 were larger than that of the aluminum tunnels (52.3 mm^2^), the grafts could be inserted to the aluminum tunnel. Therefore, we speculate that there was some pressure registered by each sensor. However, in the round tunnel, no pressure was detected at the vertical sensor. Fujii showed that the center of the graft shifted more than 1 mm inside a simulated 7.0-mm diameter round tunnel with 30 N at a graft angle of 75° (Fujii et al. 2014). In their observations, the initial shift occurred when the graft was bent in the vertical direction. Our results showed less vertical contact versus bilateral contact in the round tunnel, which indicates that the bending direction may be a reason for the initial graft shift. This finding, when considered with the results of the Fujii’s study, suggests that there is an invisible space in a round tunnel that may not be presented in a rectangular tunnel.

Several anatomical studies have reported that the femoral insertion for the ACL has an oval or semilunar shape (Petersen & Zantop 2008; Sasaki et al. 2012). Moreover, an anatomical single-bundle ACL reconstruction with an “oval” femoral tunnel (i.e., not a rounded rectangle) recently attracted attention; several surgical methods have been reported (Noh et al. 2011; Petersen et al. 2013). In our study, the cross-section of the graft was also oval. However, it is important to note an oval femoral tunnel has several disadvantages. The biggest difference between oval and rounded rectangular femoral tunnel is whether a straight line exist or not. It is more difficult to position the major axis of oval tunnel parallel to the lateral intercondylar ridge because there was no straight line. Moreover, in oval tunnel, the minor axis gets longer when the area was increased. Therefore, the oval femoral tunnel cannot increase in size without roof impingement and does not restore the flat tendon–bone junction, as described by Smigielski (Smigielski et al. 2014). To overcome these disadvantages, we developed the technique of anatomical single-bundle ACL reconstruction with a rounded rectangle femoral dilator. We have described that this technique could reestablish the flat tendon-bone junction compared with a round or oval tunnel, and may potentially reduce the graft failure rate of anatomical ACL reconstructions compared to that of non-anatomical or standard ACL reconstructions (Nakase et al. 2015). Therefore, the rounded rectangle tunnel was used in the present study.

It is well known that tunnel enlargement occurs after surgery when a soft tissue graft is used (Brown et al. 2004; Kobayashi et al. 2006). In the round femoral tunnel, Segawa et al. (Segawa et al. 2003) evaluated dynamic changes of the graft in the femoral round tunnel using a pressure sensor and demonstrated maximum contact pressure of the graft at the anterior portion of the femoral tunnel when the knee was in full extension. The authors concluded that this phenomenon explains the occurrence of bone tunnel enlargement at the anterior portion of the femoral tunnel. Moreover, Tachibana et al. (Tachibana et al. 2014) reported that the morphology at the femoral tunnel aperture changed with time after surgery as the tunnel walls translated anteriorly and distally. These results suggest that the tendon–bone junction lies at the anterior and distal zone of the tunnel. Histologically, the ACL midsubstance fibers form a narrow “direct” insertion posterior and along the lateral intercondylar ridge, which is continued by a fan-like “indirect” insertion toward the posterior femoral cartilage (Iwahashi et al. 2010; Mochizuki et al. 2014; Sasaki et al. 2012). Previous studies have suggested that direct insertion plays a major role in the mechanical link between the ligament and bone as compared with indirect insertion (Takahashi et al. 2006; Weiler et al. 2006). When considering the area of the tendon–bone junction and the location of the direct insertion, the femoral tunnel should be made just posterior to the lateral intercondylar ridge during an “anatomical” reconstruction.

Sasaki showed that the mean distance from the lateral intercondylar ridge to the border of the posterior cartilage was 10.1 mm, whereas the mean distance from direct insertion to the border of the posterior cartilage was only 4.4 mm in an anatomical study (Sasaki et al. 2012). Shino showed, arthroscopically, that a lateral intercondylar ridge running in line was consistently identified 7 to 10 mm anterior to the posterior articular cartilage margin of the lateral femoral condyle (Shino et al. 2010). These findings indicate that it is difficult to make a large-diameter “round” tunnel in an anatomical position without blowing out the posterior wall of the lateral condyle.

Because the mechanical strength of a tendon graft declines after reconstruction (Beynnon et al. 1997), a larger cross-sectional area of graft would mitigate this reduction in strength (Grood et al. 1992). A biomechanical study showed that the mean load to failure was about 2400 N, 3300 N, 3900 N, and 4400 N for 6-, 7-, 8-, and 9-mm diameter grafts, respectively (Boniello et al. 2015). Clinically, recent evidence has pointed to a higher early failure rate of hamstring autografts in patients with grafts of 8-mm diameter or less when compared with grafts greater than 8 mm in diameter (Conte et al. 2014; Magnussen et al. 2012). Magnussen et al. showed that decreased autograft hamstring size is a predictor of early graft failure in patients younger than 20 years old. At a mean follow-up of 14 months, revision was required in 1.7 % of grafts greater than 8 mm in diameter, 6.5 % of 7.5- or 8-mm grafts, and 13.6 % of grafts 7 mm or less in diameter. The variation and significantly lower failure loads in smaller grafts seen in our study would certainly substantiate these results. In particular, the failure rate of grafts 8 mm or less in diameter was 18.3 % for patients aged 18 years or younger, whereas patients older than 18 years had a failure rate of 7 %.

It is difficult to make an 8-mm diameter “round” tunnel in an anatomical position without blowing out the posterior wall of the lateral condyle given the lateral intercondylar ridge to posterior cartilage border ranges from 7 to 10 mm (Sasaki et al. 2012; Shino et al. 2010). On the other hand, we believe it is safer to create an anatomical “rounded rectangular” tunnel with an area larger than that of the 8-mm diameter “round” tunnel; it is important that the major axis of the rounded rectangular tunnel be parallel to the lateral intercondylar ridge.

It may not be straightforward to simply state which shape of tunnel is best. However, as no study to date has quantitatively assessed the difference between round and rounded rectangular tunnels, we believe that our results provide important foundational information regarding this point. The shape of the tunnel has generally been considered as round because of the round shape of the drill bit. This study revealed that the cross-sectional shape of the femoral graft was not round, but oval. Moreover, a rounded rectangular femoral tunnel was more suitably fitted for grafts than a round femoral tunnel. Additional studies need to extend these considerations of the shape of the tunnel, for example by evaluating tunnel enlargement and dynamic influences.

This study had several limitations. First, only an 8.16-mm diameter round tunnel and 6 × 10 rounded rectangular tunnel were evaluated. If a round tunnel with 7 mm diameter was assessed, there may have been contact pressure at the vertical sensor. However, the round and rounded rectangular tunnels that were evaluated had the same area (52.3 mm^2^), so the results are meaningful. Another limitation is that only seven grafts were used for the investigation. Third, the tibial side of the graft was not investigated because the stich technique influenced the cross-section and contact pressure. Fourth, the mean age of the cadaver specimens was higher than patients who undergo ACL reconstruction (i.e., there may be differences in elasticity between young and old ACL). Fifth, we use only semitendinosus. We must cut the graft to evaluate the cross-section in this method, therefore we couldn’t investigate other patterns of the graft such as using each two fold of gracilis and semitendinosus (total four fold). Finally, this study did not consider graft-bending angle and knee joint ROM, necessitating further biomechanical studies.

## Conclusions

The shape of the tunnel has generally been made as round because the shape of the drill is round, and more consideration should be given to the shape of the tunnel. The cross-sectional shape of the femoral graft was not round, but oval. In this study, a rounded rectangular femoral tunnel was more suitably fitted than a round femoral tunnel for grafts. Additional studies, such as evaluation of tunnel enlargement and dynamic influence, are required.
